# Association between antinuclear antibodies and pregnancy prognosis in recurrent pregnancy loss patients

**DOI:** 10.1093/humrep/deae280

**Published:** 2024-12-20

**Authors:** H Yoshihara, S Goto, Tamao Kitaori, M Sugiura-Ogasawara

**Affiliations:** Department of Obstetrics and Gynecology, Graduate School of Medical Sciences, Nagoya City University, Nagoya, Aichi, Japan; Department of Obstetrics and Gynecology, Graduate School of Medical Sciences, Nagoya City University, Nagoya, Aichi, Japan; Department of Obstetrics and Gynecology, Graduate School of Medical Sciences, Nagoya City University, Nagoya, Aichi, Japan; Department of Obstetrics and Gynecology, Graduate School of Medical Sciences, Nagoya City University, Nagoya, Aichi, Japan

**Keywords:** antinuclear antibodies, antinuclear antibodies titers, antinuclear antibodies patterns, recurrent pregnancy loss, pregnancy outcomes, autoimmune disorders, antiphospholipid syndrome, antiphospholipid antibodies, cohort study, predictive markers

## Abstract

**STUDY QUESTION:**

Can antinuclear antibodies (ANA) affect the subsequent live birth rate (LBR) in patients with unexplained recurrent pregnancy loss (RPL) in the absence of antiphospholipid antibodies (aPL)?

**SUMMARY ANSWER:**

Women with unexplained RPL have a high probability of live birth following a positive pregnancy test (>70%), being similar between those with positive and negative ANA testing, regardless of the cut-off value.

**WHAT IS KNOWN ALREADY:**

The RPL guidelines of the ESHRE state that ‘ANA testing can be considered for explanatory purposes’. However, there have been a limited number of studies on this issue and sample sizes have been small, and the impact of ANA on the pregnancy prognosis is unclear.

**STUDY DESIGN, SIZE, DURATION:**

A retrospective cohort study was conducted at Nagoya City University Hospital between 2006 and 2019. The study included 1021 women with RPL without known cause.

**PARTICIPANTS/MATERIALS, SETTING, METHODS:**

Hysterosalpingography or 3D-ultrasound, chromosome analysis for both partners, blood tests for aPL, ANA, hypothyroidism, and diabetes mellitus were performed before a subsequent pregnancy. ANAs were measured by indirect immunofluorescence on Hep-2 cell slides. The cutoff dilution used was 1:40. In addition, patients were classified according to the ANA pattern on immunofluorescence staining: homogeneous, speckled, nucleolar, centromeric, peripheral, cytoplasmic, and others. LBRs were compared between ANA-positive and ANA-negative patients after excluding patients with antiphospholipid antibody syndrome, an abnormal chromosome in either partner and a uterine anomaly.

**MAIN RESULTS AND THE ROLE OF CHANCE:**

Considering the cut-off value = 1:40 dilution, the subsequent LBRs were 72.5% (256/353) for the ANA-positive group and 73.2% (489/668) for the ANA-negative group; odds ratio (OR) = 0.97, 95% CI = 0.72–1.29. After excluding the miscarriages occurring from embryonic aneuploidy, the biochemical pregnancy losses, and the ectopic pregnancies, LBRs were 92.8% (256/276) for the ANA-positive group and 93.0% (489/526) for the ANA-negative group: OR = 0.97 (95% CI = 0.55–1.70). Considering the cut-off value = 1:80 dilution, the subsequent LBRs were 75.0% (87/116) for the ANA-positive group and 72.7% (658/905) for the ANA-negative group; OR = 1.13 (95% CI = 0.72–1.76). After excluding the miscarriages occurring from embryonic aneuploidy, the biochemical pregnancy losses, and the ectopic pregnancies, LBRs were 89.7% (87/97) for the ANA-positive group and 93.3% (658/705) for the ANA-negative group: OR = 0.62 (95% CI = 0.30–1.27). Considering the cut-off value = 1:160 dilution, the subsequent LBRs were 82.4% (28/34) for the ANA-positive group and 72.6% (717/987) for the ANA-negative group; OR = 1.76 (95% CI = 0.72–4.29). After excluding the miscarriages occurring from embryonic aneuploidy, the biochemical pregnancy losses, and the ectopic pregnancies, LBR were 93.3% (28/30) for the ANA-positive group and 92.9% (717/772) for the ANA-negative group: OR = 1.07 (95% CI = 0.25–4.63). There was no difference in LBR between the 2 groups before or after adjustment for age and BMI, but ANA-positive patients were significantly older than ANA-negative patients when using the 1:40 dilution, and ANA-positive patients had significantly lower BMIs than ANA-negative patients when using the 1:80 dilution.

**LIMITATIONS, REASONS FOR CAUTION:**

A healthy control group was not established, making it impossible to compare ANA positivity rates between healthy controls and RPL patients. There were significant differences in age (1:40 dilution) and BMI (1:160 dilution) between the ANA-positive and ANA-negative groups.

**WIDER IMPLICATIONS OF THE FINDINGS:**

Our results suggest that ANA testing is not useful to predict future pregnancy loss in women with RPL without known cause.

**STUDY FUNDING/COMPETING INTEREST(S):**

This study was supported by MEXT Promotion of Distinctive Joint Research Center Program, Grant Number JPMXP0621467963 and used for English proofreading costs. There are no competing interests for all authors.

**TRIAL REGISTRATION NUMBER:**

N/A.

## Introduction

Pregnancy loss is a common complication in early pregnancy. Recurrent pregnancy loss (RPL) is prevalent (1–2% of women) (Ford and Schust, 2009). Causal factors can include antiphospholipid antibody syndrome (APS), uterine anomaly, parental chromosomal abnormalities, and embryo aneuploidy, but there are still many patients with RPL whose cause is not yet understood (Sugiura-Ogasawara *et al.*, 2012). Inadequate thyroid disease management and poor glycemic control are associated with miscarriage (Abalovich *et al.*, 2002; Inkster *et al.*, 2006), although further evidence is needed on the association with RPL.

The involvement of immunological mechanisms in RPL of unknown cause has been demonstrated, and the adverse effects of autoimmune diseases have long been recognized (D’Ippolito *et al.*, 2020). The role of autoantibodies in RPL has received increasing attention in recent years.

The most common autoantibodies are antinuclear antibodies (ANA), conventionally assessed by indirect immunofluorescence, which include antibodies targeting nuclear, cytoplasmic, and mitotic patterns (Chan *et al.*, 2015). ANA positivity, expressed in low titers, is common in healthy women, but the presence of high titers (>1:160) is strongly associated with autoimmune diseases such as systemic lupus erythematosus (SLE), systemic sclerosis (SSc), and Sjogren’s syndrome (SjS), which are associated with adverse pregnancy events (Agmon-Levin *et al.*, 2014). When HEp-2 cells, the substrate for the indirect immunofluorescence assay, are permeabilized and incubated with a patient’s serum, various staining patterns are observed, including homogeneous, speckled, nucleolar, nuclear membranous, centromeric, and nuclear (Damoiseaux *et al.*, 2019). These patterns are associated with ANA subtypes and certain autoimmune diseases.

The European Society of Human Reproduction and Embryology (ESHRE) 2022 guidelines conditionally recommend the ANA test for explanatory purposes (Bender Atik *et al*., 2023). However, it is not recommended as a screening test for RPL by the American Society for Reproductive Medicine (Practice Committee of the American Society for Reproductive Medicine, 2012). A difference in the prevalence of ANA between RPL patients and healthy women has been controversial (Molazadeh *et al.*, 2014; Hefler-Frischmuth *et al.*, 2017). Studies on this issue are limited, sample sizes are small, and the impact of ANA on pregnancy prognosis and its clinical value remain unresolved. We therefore conducted the present study to evaluate whether screening of ANA should be recommended and whether ANA affect subsequent live births in patients with RPL.

## Materials and methods

### Patients with RPL

All patients were seen at Nagoya City University Hospital from 2003 to December 2022. The study included 1465 patients with a history of two or more pregnancy losses. Biochemical pregnancy losses were included based on ESHRE RPL guidelines.

Hysterosalpingography or 3D-ultrasound, chromosome analysis for both partners, antiphospholipid antibodies (aPLs) and blood tests for ANA, thyroid-stimulating hormone, free thyroxine (FT4), and diabetes test (fasting insulin and fasting glucose, if abnormal, HbA1c) were performed before a subsequent pregnancy. We used three kinds of aPLs tests: lupus anticoagulant (LA) detected by diluted activated partial thromboplastin time (APTT), LA detected by diluted Russell’s viper venom time and β2glycoprotein I-dependent anti-cardiolipin antibody (aCL), and APS was diagnosed according to the criteria of the International Congress on APS (Miyakis *et al.*, 2006). All pregnancies were followed up at Nagoya City University Hospital until 2022.

Patients with APS, an abnormal chromosome in either partner, or a uterine anomaly were excluded from the present analysis. Since some unexplained patients wished for medication, such patients were also excluded from the analysis. Patients with abnormal thyroid function or glucose metabolism who received appropriate treatment before and during pregnancy under the supervision of an endocrinologist were included in the present analysis.

The protocol was reviewed and approved by the Research Ethics Committee of Nagoya City University.

### Antinuclear antibody measurement

ANAs were measured by indirect immunofluorescence on Hep-2 cell slides. The cutoff dilutions used were 1:40, 1:80, and 1:160 of patient serum. In addition, patients were classified according to the ANA pattern on immunofluorescence staining: speckled, homogeneous, nucleolar, nucleolar, granular, centromeric, cytoplasmic, and others.

### Outcome measures

The primary outcome measure was the subsequent live birth rate (LBR) after excluding pregnancy losses caused by aneuploidy, biochemical pregnancy, ectopic pregnancy, and losses in which the cause was unknown. Live births included both term and preterm births at our hospital. Additionally, ongoing pregnancies beyond 10 weeks of gestation were also counted when patients, at their request, were transferred to local obstetric clinics for delivery and follow-up care. Gestational age was calculated from basal body temperature charts or crown–rump length or embryo transfer day. A transvaginal ultrasound was performed once a week before 10 weeks’ gestation and twice a month after 10 weeks’ gestation. When a miscarriage was diagnosed, a dilatation and curettage was performed, and cytogenetic analysis of products of miscarried conception (POC) was carried out. Losses in which the cause was unknown include cases where chromosome testing of POC was not possible due to complete miscarriage and cases where the patient refused testing because it was self-funded. LBRs were compared between ANA-positive and ANA-negative patients.

The secondary measures were the frequency of pregnancy losses with a normal embryonic or fetal karyotype, aneuploidy, or an unknown cause and the frequency of a chemical pregnancy, an ectopic pregnancy, or a preterm birth at <34 weeks’ gestation.

### Statistical analysis

Demographic characteristics were summarized using means with standard deviation for parametric continuous data and counts expressed as a percentage (%) for discrete data. Baseline differences between groups were analyzed using chi-square tests for categorical variables and Mann–Whitney *U*-tests for continuous data. Chi-square tests were also used to compare the LBR and subsequent pregnancy and perinatal outcomes. Binomial logistic regression analysis was performed on the LBR to calculate adjusted odds ratios (ORs) and 95% CI between the ANA-positive and ANA-negative patients. The analyses were adjusted for age, BMI, the number of previous early miscarriages, the number of previous live births, and the presence of IVF-ET. Analyses were performed using SPSS Windows Version 22.0 (SPSS Japan Inc., Tokyo, Japan). *P* < 0.05 or a two-sided 95% CI of ratio excluding one was considered to be statistically significant.

## Results

**Figure 1.**
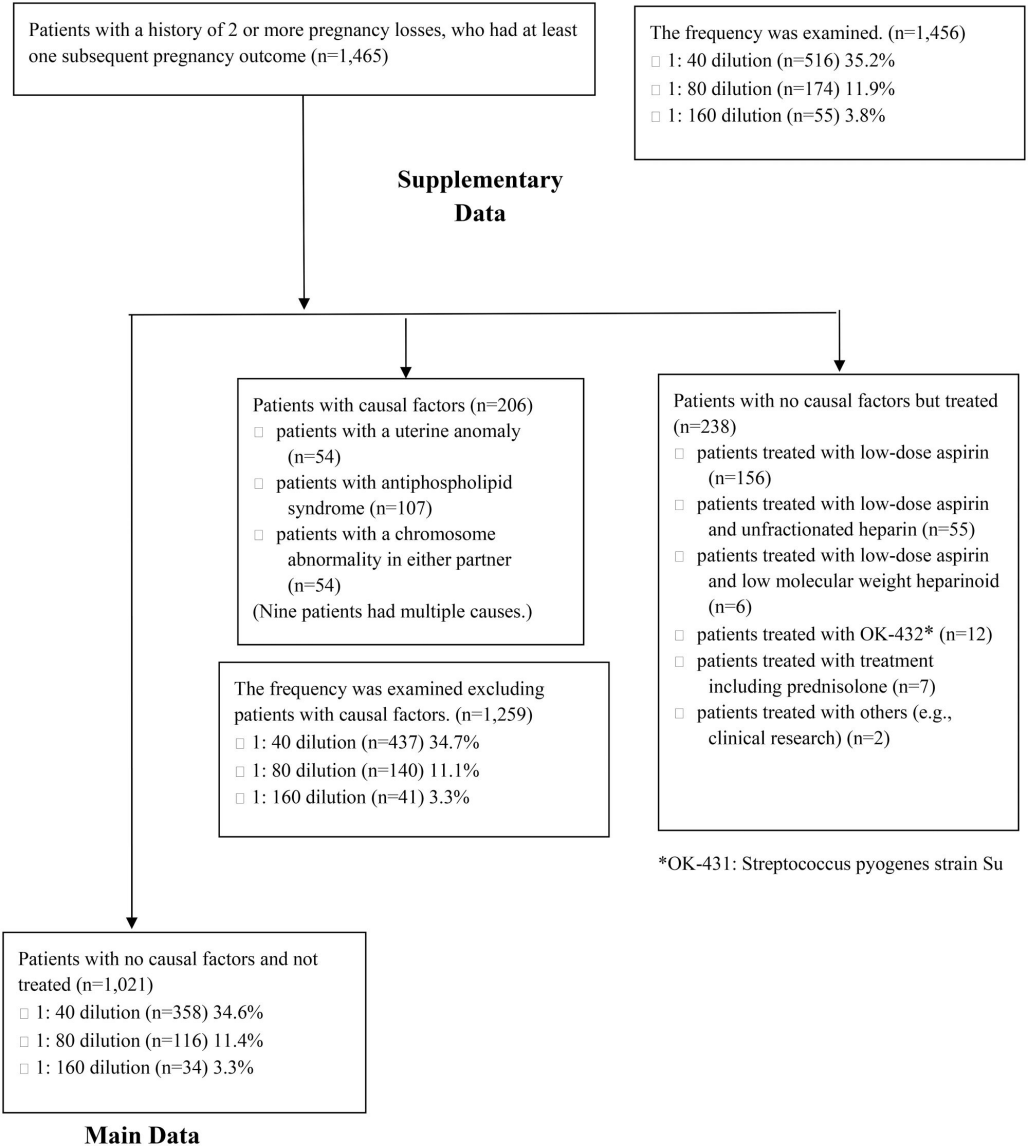
**The flow diagram**.

The study included 1465 patients with a history of 2 or more pregnancy losses on RPL evaluation. The characteristics of the 1465 patients and the subsequent live births rates are shown in Supplementary Tables S1 and S2. The rate of ANA-positive patients was 35.2% (516/1465) when the 1:40 dilution result was positive. With a 1:80 dilution, the rate of ANA-positive patients was 11.9% (174/1465). With a 1:160 dilution, the rate of ANA-positive patients was 3.8% (55/1465). Age, number of previous early miscarriages, the presence of causal factors such as APS or uterine anomaly, and parental chromosomal abnormalities were identified as factors defining next pregnancy outcome (Supplementary Table S3). Patients were categorized into those with causal factors, those with no causal factors but treated, and those with no causal factors and not treated (Fig. 1). Thus, further analyses were performed in untreated patients without causal factors.

The characteristics of the 1021 patients with no causal factors and not treated are shown in Table 1. When using the 1:40 dilution, the median age was 33 years (interquartile range (IQR), 31–38) in the ANA-positive group and 33 years (IQR, 30–37) in the ANA-negative group (*P* = 0.039). When using the 1:160 dilution, the median BMI was 19.8 (IQR, 18.1–20.9) and 20.4 (IQR, 19.1–22.3), respectively (*P* = 0.027). There were no significant differences in age or BMI between the two groups when using 1:80 dilution. The median number of previous prior losses was 2 (IQR, 2–3), respectively, and there were no significant differences between the two groups at any dilution.

**Table 1.** Characteristics of patients with recurrent pregnancy loss with no known cause and receiving no treatment.

		All N=1021	Antinuclear antibody cut-off value = 1:40		*P* *	Antinuclear antibody cut-off value = 1:80		*P* *	Antinuclear antibody cut-off value = 1:160		*P* *
			Positive N=353	Negative N=668		Positive N=116	Negative N=905		Positive N=34	Negative N=987	
Age at pregnancy (years)	Median (IQR)	33.0 (30.0, 37.0)	33.0 (31.0, 38.0)	33.0 (30.0, 37.0)	0.039	33.0 (30.0, 37.0)	33.0 (31.0, 37.0)	1.00	33.0 (31.0, 37.0)	33.0 (30.0, 37.0)	0.98
BMI (kg/m²)	Median (IQR)	20.4 (19.0, 22.2)	20.6 (19.1, 22.2)	20.3 (19.0, 22.3)	0.44	20.4 (19.1, 22.1)	20.4 (19.0, 22.3)	0.78	19.8 (18.1, 20.9)	20.4 (19.1, 22.3)	0.027
Prior total pregnancy losses	Median (IQR)	2.0 (2.0, 3.0)	2.0 (2.0, 3.0)	2.0 (2.0, 3.0)	0.91	2.0 (2.0, 3.0)	2.0 (2.0, 3.0)	0.51	2.0 (2.0, 3.0)	2.0 (2.0, 3.0)	0.27
Prior early miscarriages	Median (IQR)	2.0 (2.0, 3.0)	2.0 (2.0, 3.0)	2.0 (2.0, 3.0)	0.53	2.0 (2.0, 3.0)	2.0 (2.0, 3.0)	0.67	2.0 (2.0, 3.0)	2.0 (2.0, 3.0)	0.57
Prior early miscarriages	0	1.1% (11)	0.8% (3)	1.2% (8)	0.45	0.8% (3)	1.1% (10)	0.96	0.0% (0)	1.1% (11)	0.71
	1	6.0% (61)	6.8% (24)	5.5% (37)		6.8% (24)	6.0% (54)		8.8% (3)	5.9% (58)	
	2	58.2% (594)	59.2% (209)	57.6% (385)		59.2% (209)	58.3% (528)		61.8% (21) 20.6% (7)	58.1% (573)	
	3	28.6% (292)	25.8% (91)	30.1% (201)		25.8% (91)	28.6% (259)		8.8% (3)	28.9% (285)	
	4 or more	6.2% (63)	7.4% (26)	5.5% (37)		7.3% (26)	6.0% (54)			6.1% (60)	
Prior late miscarriages and stillbirths	0	95.7%	94.9%	96.1%	0.59	94.0%	95.9%	0.62	97.1% (33)	95.6%	0.87
	1	(977)	(335)	(642)		(109)	(868)		2.9% (1)	(944)	
	2 or more	3.7% (38)	4.2% (15)	3.4% (23)		5.2% (6)	3.5% (32)		0.0% (0)	3.7% (37)	
		0.6% (6)	0.8% (3)	0.4% (3)		0.9% (1)	0.6% (5)			0.6% (6)	
Prior biochemical pregnancy losses	0	87.3%	85.8%	88.0%	0.60	87.9%	87.2%	0.96	88.2% (30)	87.2%	0.98
	1	(891)	(303)	(588)		(102)	(789)		8.8% (3)	(861)	
	2 or more	9.3% (95)	10.5% (37)	8.7% (58)		8.6% (10)	9.4% (85)		2.9% (1)	9.3% (92)	
		3.5% (35)	3.7% (13)	3.3% (22)		3.4% (4)	3.4% (31)			3.4% (34)	
Prior live births	1 or more	21.4% (218)	21.2% (75)	21.4% (143)	1.00	21.6% (25)	21.3% (193)	1.00	23.5% (8)	21.3% (210)	0.83
IVF-ET	Presence	7.4% (76)	5.9% (21)	8.2% (55)	0.21	4.3% (5)	7.8% (71)	0.26	2.9% (1)	7.6% (75)	0.51

* Baseline differences between antinuclear antibody positive and negative groups were analyzed using chi-square tests for categorical variables and Mann–Whitney *U*-tests for continuous data.

The subsequent LBR was calculated for the 1021 patients (Table 2). With the use of the 1:40 dilution, the subsequent LBRs were 72.5% (256/353) for the ANA-positive group and 73.2% (489/668) for the ANA-negative group. After excluding miscarriages with embryonic aneuploidy, biochemical pregnancies and ectopic pregnancies and those of unknown cause, LBRs were 92.8% (256/276) for the ANA-positive group and 93.0% (489/526) for the ANA-negative group. No significant difference is observed (Table 2).

**Table 2.** Subsequent pregnancies and perinatal outcomes in 1021 patients with recurrent pregnancy loss who were antinuclear antibody (ANA) positive or negative.

	1:40 dilution		Crude OR (95% CI)*	1:80 dilution		Crude OR (95% CI)*	1:160 dilution		Crude OR (95% CI)*
	Positive	Negative		Positive	Negative		Positive	Negative	
Number	353	668		116	905		34	987	
Live births	72.5% (256/353)	73.2% (489/668)	0.97 (0.72–1.29)	75.0% (87/116)	72.7% (658/905)	1.13 (0.72–1.75)	82.4% (28/34)	72.6% (717/987)	1.76 (0.72–4.29)
Live births after excluding abnormal karyotypes^†^	92.8% (256/276)	93.0% (489/526)	0.97 (0.55–1.70)	89.7% (87/97)	93.3% (658/705)	0.62 (0.30–1.27)	93.3% (28/30)	92.9% (717/772)	1.07 (0.25–4.63)
Pregnancy losses	27.5% (97/353)	26.8% (179/668)		25.0% (29/116)	27.0% (247/916)		17.6% (6/34)	27.4% (270/987)	
Normal karyotypes	20.6% (20)	20.7% (37)		34.5% (10)	18.9% (47)		33.3% (2)	20.4% (55)	
Abnormal karyotypes	35.1% (34)	38.0% (68)		27.6% (8)	38.1% (94)		33.3% (2)	37.0% (100)	
Chemical pregnancies	15.5% (15)	17.9% (32)		13.8% (4)	17.4% (43)		33.3% (2)	16.7% (45)	
Ectopic pregnancies	2.1% (2)	1.7% (3)		3.4% (1)	1.6% (4)		0.0% (0)	1.9% (5)	
Unknown causes^‡^	26.8% (26)	21.8% (39)		20.7% (6)	23.9% (59)		0.0% (0)	24.1% (65)	
Preterm births <34 weeks gestation^§^	0.8% (1/131)	2.0% (6/296)	0.37 (0.44–3.12)	0.0% (0/48)	1.9% (7/379)	0.98 (0.97–1.00)	0.0% (0/15)	1.7% (7/412)	0.98 (0.97–1.00)
Birth weights (g)^¶^	3072 (130)	2986 (292)		2988 (47)	3015 (375)		2999 (15)	3013 (407)	

Three standards were examined: 1:40, 1:80, and 1:160 dilutions.

* Chi-square tests were performed.

† Excluding abnormal karyotypes, chemical pregnancies, ectopic pregnancies, and cases of unknown origin.

‡ Cases of complete miscarriage or chorionic chromosome test not requested.

§ Patients whose gestational weeks at delivery were unknown were excluded from the denominator.

¶ Mean (SD).

With the use of the 1:80 dilution, the subsequent LBRs were 75.0% (87/116) for the ANA-positive group and 72.7% (658/905) for the ANA-negative group. After excluding miscarriages with embryonic aneuploidy, biochemical pregnancies and ectopic pregnancies and those of unknown cause, LBRs were 89.7% (87/97) for the ANA-positive group and 93.3% (658/705) for the ANA-negative group. No significant difference is observed (Table 2).

Using the 1:160 dilution, the subsequent LBRs were 82.4% (28/34) for the ANA-positive group and 72.6% (717/987) for the ANA-negative group. After excluding miscarriages with embryonic aneuploidy, biochemical pregnancies, and ectopic pregnancies and those of unknown cause, LBRs were 93.3% (28/30) for the ANA-positive group and 92.9% (717/772) for the ANA-negative group. No significant difference is observed (Table 2).

Subgroup analyses were performed for each pattern on immunofluorescence staining, but there was no significant difference in the proportion of staining types between the live birth and miscarriage groups (Table 3). Duplicate speckled and homogenous patterns were seen in 21.1% of all cases. Around 70% of the ANA-positive group had multiple ANA staining patterns.

**Table 3.** Prevalence of antinuclear antibody staining patterns and subsequent pregnancy outcomes among patients with recurrent pregnancy loss.

Antinuclear antibody staining patterns	All N = 1021. Percentage of total patients (number of patients)	Subsequent pregnancy outcome		***P*** *
		Live birth (N = 753). Percentage of patients with live birth (number of patients)	Pregnancy loss (N = 279). Percentage of patients with pregnancy loss (number of patients)	
Speckled and homogeneous	21.1% (215)	20.8% (155)	21.7% (60)	
Speckled	8.8% (90)	8.5% (63)	9.8% (27)	
Speckled and nucleolar	1.3% (13)	1.3% (10)	1.1% (3)	
Speckled and granular	0.7% (7)	0.8% (6)	0.4% (1)	
Speckled and homogenous and granular	0.6% (6)	0.7% (5)	0.4% (1)	
Homogeneous	0.5% (5)	0.5% (4)	0.4% (1)	
Nucleolar	0.4% (4)	0.3% (2)	0.7% (2)	
Speckled and cytoplasmic	0.3% (3)	0.4% (3)	0% (0)	
Speckled and homogeneous and nucleolar	0.3% (3)	0.3% (2)	0.4% (1)	
Granular	0.2% (2)	0.3% (2)	0% (0)	
Other staining patterns	0.6% (6)	0.7% (5)	0.4% (1)	
Negative	65.3% (667)	65.5% (488)	64.9% (179)	0.97

* Chi-square tests were performed between the live birth group and the pregnancy loss group.

**Figure 2.**
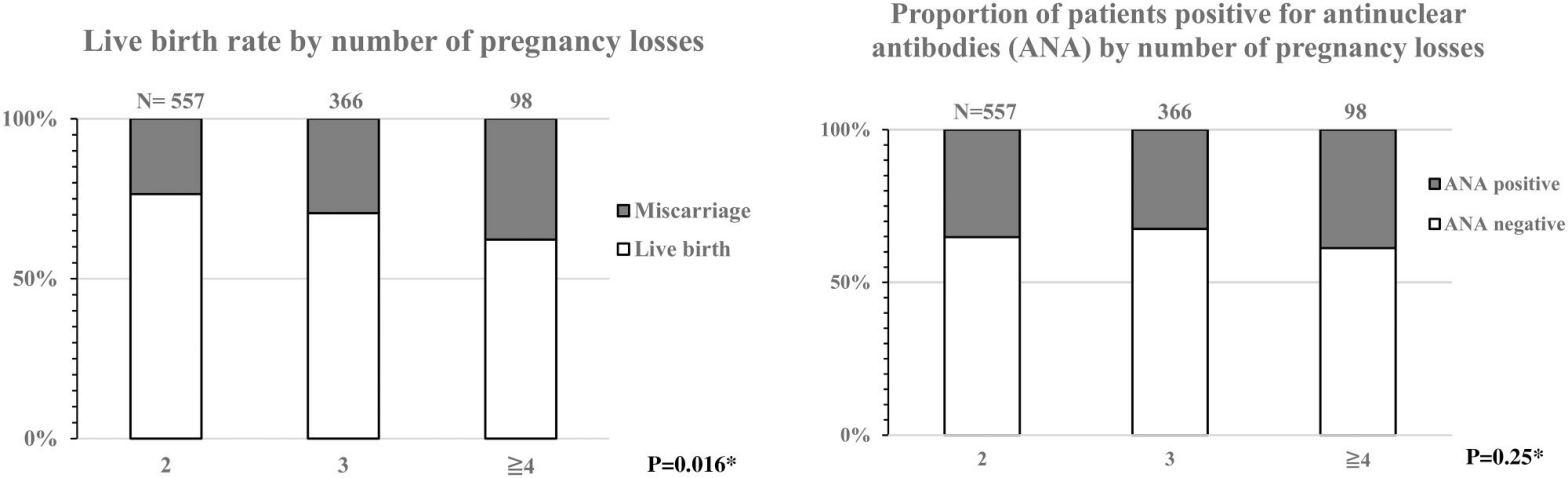
**Live birth rate by number of miscarriages and proportion of patients positive for antinuclear antibodies by number of miscarriages.** This included patients from the main data (n = 1021). *Pearson’s chi-square test was conducted.

The prevalence of APS was significantly higher in patients with ANA-positive than patients with ANA-negative (Supplementary Table S4). The rate of live births decreased with the number of miscarriages (*P* = 0.024), while the proportion of patients positive for ANA remained unchanged (*P* = 0.714) (Fig. 2).

## Discussion

To the best of our knowledge, this study reports the highest total number of RPL women collected to date in relation to ANA. We have already reported that the frequency of ANA positivity was significantly higher in 225 RPL patients with a history of 2 miscarriages than in 740 healthy pregnant women, but there was no difference in subsequent LBR between 39 ANA positive and 186 negative patients (Ogasawara *et al.*, 1996). In contrast, Cavalcante *et al.* (2015) reported that ANA positivity was more common in RPL patients who had a subsequent miscarriage (n = 24) compared to those who had a live birth (n = 82) when treated with lymphocyte immunotherapy. A small prospective study reported a higher miscarriage rate in ANA-positive women compared to RPL ANA-negative women (Harger *et al.*, 1983).

We examined whether ANA predicts the next pregnancy prognosis for 1021 patients with RPL; however, we found no predictive value of ANA. A recent meta-analysis showed a significant association with RPL, particularly in patients with high-titer ANA positivity >160-fold (Chen *et al.*, 2020). However, in our study results, even ANA positivity at high titers of 160-fold or more was not associated with the outcome of the next pregnancy in patients with unexplained and untreated RPL. Probability of live birth is known to decrease in a dose-dependent manner with increasing history of miscarriage and maternal age, although they are not sufficient to predict whether a pregnancy will result in a live birth (Kolte *et al.*, 2021). The present study found similar results, with birth rate decreasing with the number of miscarriages.

It is well known that the frequency of ANA positivity increases with age and that its prevalence is higher in individuals with lower BMI (Satoh *et al.*, 2012). In this study, using a 1:40 dilution, ANA-positive patients were found to be significantly older than ANA-negative patients, while using a 1:160 dilution, ANA-positive patients had significantly lower BMI than ANA-negative patients. However, no significant difference in birth rate was observed between the two groups. Differences in age and BMI may serve as confounding factors and limitations in this study. Therefore, we adjusted for age, BMI, and other explanatory variables; even after adjustment, we did not observe a significant difference in birth rate between the two groups (Supplementary Table S4). In the present analysis, the highest multiplier for ANA was 1280 times, and all three of them had achieved live births. These indicate that ANA is not a direct cause of RPL. Immunosuppressant drugs should not be considered solely because of positive ANA.

Some of the ANA-positive populations are thought to represent the preclinical stage of autoimmune disease, based on the observation that autoantibodies are usually produced prior to the clinical manifestations of the disease (Arbuckle *et al.*, 2003). Immunostaining patterns are associated with specific autoantibodies, such as homogenous found in patients with SLE, fine speckled found in patients with SjS and SLE and centromere found in SSc and may be useful in diagnosing collagen disease (Damoiseaux *et al.*, 2019). In SLE, the entry criterion is 80-fold, and the diagnosis is made using the pattern of ANA as a clue, as well as the detection of specific autoantibodies, which do not reflect disease status (Pisetsky *et al.*, 2019). Although a recent meta-analysis showed more homogenous patterns of ANA in the RPL group, in our study, there were no staining patterns that would determine the next pregnancy prognosis for RPL (Chen *et al.*, 2020).

Analysis of 1128 patients with APS and without known factors showed that ANA-positive patients had a higher rate of being diagnosed with APS (Supplementary Table S5: OR: 1.60, 95% CI: 1.07–2.39). Patients with APS, especially those with positive LA, are known to have a poor next pregnancy prognosis if untreated (Clark *et al.*, 2013). ASRM, ESHRE guidelines, and the Lancet series recommend the use of classification criteria by the International Congress on antiphospholipid antibodies (Miyakis *et al.*, 2006; Molazadeh *et al.*, 2014; Hefler-Frischmuth *et al.*, 2017; Quenby *et al.*, 2021). However, recently, a new criterion was developed by the American College of Rheumatology and the European Alliance of Associations for Rheumatology (Barbhaiya *et al.*, 2023). The methods for diagnosing obstetric APS have not been confirmed to improve LBR in patients with RPL. If APS patients are not properly diagnosed and treated, ANA-positive RPL patients may have a poor pregnancy prognosis. ANA have been reported to cause complement activation and increased immune complexes (Veglia *et al.*, 2015), but the mechanism may not be due to ANA but to LA. Possible reasons for the high frequency of ANA positivity in RPL patients include the presence of fetal cells in maternal blood and high levels of estrogen, progesterone, and prolactin (Satoh *et al.*, 2012).

With regard to the ANA positivity rate, the current study found 34.6% at 40×, 11.2% at 80×, and 3.39% at 160× or more. A recent meta-analysis reported cut-offs of 23.68% (698/2947) for RPL women and 7.63% (240/3145) for control women, although cut-offs varied between studies (Ticconi *et al.*, 2023). These differences may be related to differences in the populations studied and variations in ANA assessment between laboratories. A limitation of our study is that a healthy control group was not established.

We conclude that women with RPL without APS, uterine anomaly or chromosome abnormality have a high probability of live birth following a positive pregnancy test, being similar between those with positive and negative ANA testing, regardless of the cut-off value and that measurement of ANA is not necessary in women with RPL to predict next pregnancy prognosis.

## Supplementary Material

deae280_Supplementary_Table_S1

deae280_Supplementary_Table_S2

deae280_Supplementary_Table_S3

deae280_Supplementary_Table_S4

deae280_Supplementary_Table_S5

## Data Availability

Data are not publicly available. However, on reasonable request, data can be obtained from the corresponding author.
