# Supplementary material for: Association between antinuclear antibodies and pregnancy prognosis in recurrent pregnancy loss patients
Source: Hum Reprod. 2024 Dec 20;40(2):236–43. doi: 10.1093/humrep/deae280 (PMC11788191; doi:10.1093/humrep/deae280)
Supplement: deae280_Supplementary_Table_S1 [file deae280_supplementary_table_s1.pdf]

Human Reproduction, 2024, 1–1
https://doi.org/10.1093/humrep/deae280
Supplementary Data

Supplementary Table S1. Characteristics of patients according to antinuclear antibody status

			Antinuclear antibody[1]		
		**All N = 1465**	**Positive N=516**	**Negative N=949**	**P**
Age at pregnancy (year)	Median (IQR)	33.0 (30.0, 37.0)	33.0 (31.0, 37.0)	33.0 (30.0, 37.0)	0.30
BMI (kg/m^2)	Median (IQR)	20.6 (19.1, 22.6)	20.7 (19.1, 22.4)	20.6 (19.1, 22.8)	0.81
Prior total pregnancy losses	Median (IQR)	2.0 (2.0, 3.0)	2.0 (2.0, 3.0)	2.0 (2.0, 3.0)	0.67
Prior early miscarriages	Median (IQR)	2.0 (2.0, 3.0)	2.0 (2.0, 3.0)	2.0 (2.0, 3.0)	0.74
Prior early miscarriages	0	1.6% (23)	1.4% (7)	1.7% (16)	0.64
	1	7.3% (107)	7.4% (38)	7.3% (69)	
	2	53.3% (781)	54.7% (282)	52.6% (499)	
	3	30.0% (439)	28.1% (145)	31.0% (294)	
	4 or more	7.9% (115)	8.5% (44)	7.5% (71)	
Prior late miscarriages and stillbirths	0	93.0% (1363)	92.6% (478)	93.3% (885)	0.56
	1	5.7% (83)	6.0% (31)	5.5% (52)	
	2 or more	1.3% (19)	1.4% (7)	1.3% (12)	
Prior biochemical pregnancy losses	0	87.2% (1277)	85.7% (442)	88.0% (835)	0.73
	1	9.4% (138)	10.7% (55)	8.7% (83)	
	2 or more	3.4% (48)	3.7% (19)	3.2% (31)	
Prior live births	1 or more	20.7% (303)	20.7% (107)	20.7% (196)	0.19
IVF-ET	Presence	8.5% (124)	8.5% (44)	8.4% (80)	1.00

[1] Based on 1/40 dilution of patient serum. Baseline differences between antinuclear antibody positive and negative groups were analyzed using chi-square tests for categorical variables and Mann–Whitney *U*-tests for continuous data.
