# Supplementary material for: Association between antinuclear antibodies and pregnancy prognosis in recurrent pregnancy loss patients
Source: Hum Reprod. 2024 Dec 20;40(2):236–43. doi: 10.1093/humrep/deae280 (PMC11788191; doi:10.1093/humrep/deae280)
Supplement: deae280_Supplementary_Table_S2 [file deae280_supplementary_table_s2.pdf]

OXFORD
human reproduction

Supplementary Table S2. Subsequent pregnancies and perinatal outcomes in 1465 patients with recurrent pregnancy loss who were antinuclear antibody (ANA) positive or negative.

	1:40 dilution			1:80 dilution			1:160 dilution		
	**Positive**	**Negative**	**Crude OR (95% CI)***	**Positive**	**Negative**	**Crude OR (95% CI)***	**Positive**	**Negative**	**Crude OR (95% CI)***
Number	516	949		174	1291		55	1410	
Live births	68.6% (354/516)	72.7% (690/949)	0.82 (0.65–1.08)	68.4% (119/174)	71.6% (925/1291)	0.86 (0.61–1.21)	70.9% (39/55)	71.3% (1005/1410)	0.98 (0.54–1.78)
Preterm births <34 weeks gestation†	0.5% (1/189)	2.2% (9/409)	0.24 (0.30–1.88)	0.0% (0/67)	1.9 % (10/531)	0.98 (0.97–0.99)	0.0% (0/22)	1.7% (10/576)	0.98 (0.97–0.99)

Three standards were examined: 1:40, 1:80, and 1:160 dilutions.
* Chi-square tests were performed.
† Patients whose gestational weeks at delivery were unknown were excluded from the denominator.

Human Reproduction, 2024, 1–1
https://doi.org/10.1093/humrep/deae280
Supplementary Data
