# Supplementary material for: Association between antinuclear antibodies and pregnancy prognosis in recurrent pregnancy loss patients
Source: Hum Reprod. 2024 Dec 20;40(2):236–43. doi: 10.1093/humrep/deae280 (PMC11788191; doi:10.1093/humrep/deae280)
Supplement: deae280_Supplementary_Table_S3 [file deae280_supplementary_table_s3.pdf]

OXFORD
human reproduction
Human Reproduction, 2024, 1–1
https://doi.org/10.1093/humrep/deae280
Supplementary Data

Supplementary Table S3. Binominal logistic regression analysis for achieving live birth.

**All N = 1465**	**Odds ratio**	**95% CI**	**P**
Age at pregnancy	0.96	0.93, 0.98	**0.001**
BMI	0.99	0.95, 1.03	0.53
Number of previous early miscarriages	0.84	0.74, 0.96	**0.011**
Prior live births	0.99	0.77, 1.28	0.94
Antinuclear antibody	0.88	0.69, 1.12	0.30
Causal factors	0.63	0.45, 0.88	**0.007**
Antiphospholipid syndrome (n = 107)	0.81	0.52, 1.25	0.34
Uterine anomaly (n = 54)	0.42	0.23, 0.77	**0.006**
Parental chromosome abnormality (n = 54)	0.56	0.30, 1.06	0.075

P-values and odds ratios in bold represent statistically significant results ($P < 0.05$).
