# Supplementary material for: Association between antinuclear antibodies and pregnancy prognosis in recurrent pregnancy loss patients
Source: Hum Reprod. 2024 Dec 20;40(2):236–43. doi: 10.1093/humrep/deae280 (PMC11788191; doi:10.1093/humrep/deae280)
Supplement: deae280_Supplementary_Table_S4 [file deae280_supplementary_table_s4.pdf]

OXFORD
human reproduction

Human Reproduction, 2024, 1–1
https://doi.org/10.1093/humrep/deae280
Supplementary Data

Supplementary Table S4. Adjusted odds ratios (OR) for live birth rates based on antinuclear antibody (ANA) cut-off dilutions.

ANA. The cut-off dilution	All cases (N = 1021). Adjusted OR (95% CI)[#]	Excluding abnormal karyotypes.[†] Adjusted OR (95% CI)[#]
**1:40**	1.00 (0.75–1.34)	1.08 (0.61–1.90)
**1:80**	0.88 (0.56–1.38)	1.59 (0.77–3.29)
**1:160**	0.55 (0.23–1.36)	0.97 (0.22–4.21)

[#] Adjusted OR for live birth rates are presented for three ANA cut-off dilutions: 1:40, 1:80, and 1:160. Results are shown for all cases and for cases excluding abnormal karyotypes, adjusted for age, BMI, the number of previous early miscarriages, the number of previous live births, and the presence of IVF-ET.
[†] Excluding abnormal karyotypes, chemical pregnancies, ectopic pregnancies and cases of unknown origin.
