# Supplementary material for: Association between antinuclear antibodies and pregnancy prognosis in recurrent pregnancy loss patients
Source: Hum Reprod. 2024 Dec 20;40(2):236–43. doi: 10.1093/humrep/deae280 (PMC11788191; doi:10.1093/humrep/deae280)
Supplement: deae280_Supplementary_Table_S5 [file deae280_supplementary_table_s5.pdf]

OXFORD

human reproduction

Human Reproduction, 2024, 1–1
https://doi.org/10.1093/humrep/deae280
Supplementary Data

Supplementary Table S5. Higher frequency of antiphospholipid syndrome in antinuclear antibody positive patients.

	Antinuclear antibody			
**Statistics related to antiphospholipid syndrome (APS)**	**Positive (N=402)**	**Negative (N=726)**	**P[#]**	**OR**
Prevalence of APS in the total of patients with APS and without known factors (N = 1128*)	9.5% (49/402)	6.1% (58/726)	**0.026**	**1.60 (1.07–2.39)**
Live birth rate in patients with APS (N=107)	61.2% (30/49)	74.1% (43/58)	0.211	0.55 (0.24–1.25)

* This number included patients without causative factors (n = 1021) and patients with antiphospholipid syndrome (n=107).
# Chi-square tests were performed.
P-values and odds ratios in bold represent statistically significant results ($P < 0.05$), with odds ratios not crossing 1 within the 95% confidence interval.
